# Supplementary material for: A Likelihood Approach for Real-Time Calibration of Stochastic Compartmental Epidemic Models
Source: PLoS Comput Biol. 2017 Jan 17;13(1):e1005257. doi: 10.1371/journal.pcbi.1005257 (PMC5240920; doi:10.1371/journal.pcbi.1005257)
Supplement: S1 File — (TAR.GZ) [file pcbi.1005257.s014.tar.gz › HSPH_Online-SI-Revision/Additions/PF09/n100-extreme-SEITR/n100-extreme-SEITR_PF2_table-4.pdf]

1	newly 3 weeks spec	13598.	4554.41	{4425., 4653.}	66.5068	0.475113	newly 3 weeks cumul	31455.	11215.4	{11025., 11380.}	64.3445	0.447906	newly 5 weeks spec	12085.	5783.84	{5642., 5935.}
2	newly 3 weeks spec	17235.	4839.43	{4308., 5318.}	71.9209	0.557692	newly 3 weeks cumul	29177.	9371.61	{8103., 10286.}	67.8801	0.498686	newly 5 weeks spec	17453.	9158.73	{8795., 9701.}
3	newly 3 weeks spec	14424.	6668.1	{3467., 8704.}	53.7708	0.359052	newly 3 weeks cumul	24222.	11960.3	{6976., 15337.}	50.6222	0.325416	newly 5 weeks spec	18266.	12142.2	{7377., 14524.}
4	newly 3 weeks spec	13822.	7318.95	{7017., 7679.}	47.0485	0.276391	newly 3 weeks cumul	25848.	15144.7	{14468., 15915.}	41.4085	0.232479	newly 5 weeks spec	16022.	10523.5	{10273., 10748.}
5	newly 3 weeks spec	22578.	6050.34	{2856., 8385.}	73.2025	0.603618	newly 3 weeks cumul	36477.	10704.1	{5502., 14601.}	70.6553	0.558614	newly 5 weeks spec	16920.	11797.5	{6482., 14378.}
6	newly 3 weeks spec	20143.	7513.42	{4795., 9889.}	62.6996	0.451519	newly 3 weeks cumul	34123.	14310.	{9363., 18456.}	58.0636	0.398041	newly 5 weeks spec	17405.	11567.4	{8725., 13810.}
7	newly 3 weeks spec	12108.	5025.92	{4909., 5143.}	58.4909	0.381928	newly 3 weeks cumul	28975.	12428.3	{12241., 12576.}	57.1067	0.367636	newly 5 weeks spec	11332.	6273.34	{6137., 6410.}
8	newly 3 weeks spec	16980.	6675.7	{6390., 6924.}	60.6849	0.40567	newly 3 weeks cumul	32632.	13480.7	{12874., 13971.}	58.6886	0.38417	newly 5 weeks spec	14766.	10399.4	{10168., 10650.}
9	newly 3 weeks spec	23304.	1639.91	{443., 2818.}	92.963	1.2532	newly 3 weeks cumul	35304.	3168.12	{1092., 5159.}	91.0262	1.12604	newly 5 weeks spec	17411.	3875.97	{707., 7052.}
10	newly 3 weeks spec	12030.	6952.35	{6572., 7222.}	42.2082	0.238383	newly 3 weeks cumul	25011.	15017.1	{14163., 15519.}	39.958	0.221758	newly 5 weeks spec	13614.	9619.24	{9233., 9889.}
11	newly 3 weeks spec	13896.	3828.23	{3693., 3956.}	72.4508	0.56027	newly 3 weeks cumul	26326.	8248.05	{8042., 8449.}	68.6696	0.504359	newly 5 weeks spec	14675.	6528.48	{6323., 6721.}
12	newly 3 weeks spec	16108.	7312.17	{6942., 7536.}	54.6054	0.343206	newly 3 weeks cumul	33166.	15763.2	{14977., 16170.}	52.4718	0.323214	newly 5 weeks spec	14159.	9924.49	{9519., 10160.}
13	newly 3 weeks spec	15592.	5840.87	{5689., 5998.}	62.5393	0.426534	newly 3 weeks cumul	30732.	12097.1	{11819., 12397.}	60.6369	0.404981	newly 5 weeks spec	14704.	9291.61	{9081., 9546.}
14	newly 3 weeks spec	10081.	3193.34	{3084., 3311.}	68.3232	0.499427	newly 3 weeks cumul	23444.	7900.47	{7736., 8069.}	66.3007	0.472443	newly 5 weeks spec	11642.	4290.43	{4126., 4460.}
15	newly 3 weeks spec	14236.	2448.95	{2351., 2547.}	82.7975	0.764614	newly 3 weeks cumul	30841.	5943.67	{5807., 6089.}	80.728	0.715146	newly 5 weeks spec	13230.	3539.18	{3407., 3691.}
16	newly 3 weeks spec	11254.	5320.99	{5194., 5461.}	52.7191	0.325413	newly 3 weeks cumul	24092.	12345.5	{12159., 12535.}	48.7568	0.290419	newly 5 weeks spec	12953.	7243.59	{7086., 7403.}
17	newly 3 weeks spec	15615.	4392.91	{2434., 7183.}	71.8674	0.599613	newly 3 weeks cumul	24517.	7914.24	{4754., 12526.}	67.7194	0.530563	newly 5 weeks spec	20574.	9209.18	{5483., 13823.}
18	newly 3 weeks spec	19007.	2702.41	{1355., 3779.}	85.782	0.883126	newly 3 weeks cumul	31170.	5216.63	{2944., 7054.}	83.2639	0.804911	newly 5 weeks spec	17189.	5999.1	{3051., 8363.}
19	newly 3 weeks spec	12889.	3515.18	{3294., 3666.}	72.7273	0.564766	newly 3 weeks cumul	24953.	7860.2	{7290., 8149.}	68.5	0.502141	newly 5 weeks spec	14542.	5672.89	{5472., 5852.}
20	newly 3 weeks spec	19368.	6435.26	{4555., 9790.}	66.7738	0.502779	newly 3 weeks cumul	33454.	12291.	{8748., 18280.}	63.2601	0.455733	newly 5 weeks spec	16864.	10552.2	{8455., 13831.}
21	newly 3 weeks spec	19672.	5019.47	{2550., 7261.}	74.4842	0.638825	newly 3 weeks cumul	30439.	8839.06	{4683., 12621.}	70.9614	0.574881	newly 5 weeks spec	18543.	10509.2	{6387., 13957.}
22	newly 3 weeks spec	14910.	8695.9	{8392., 8961.}	41.6774	0.234333	newly 3 weeks cumul	31688.	19176.2	{18433., 19640.}	39.4844	0.218278	newly 5 weeks spec	14003.	10577.2	{10383., 10780.}
23	newly 3 weeks spec	16317.	9291.98	{9086., 9497.}	43.0534	0.244597	newly 3 weeks cumul	34910.	20926.1	{20192., 21487.}	40.0569	0.222366	newly 5 weeks spec	13260.	10579.3	{10391., 10753.}
24	newly 3 weeks spec	16757.	1740.63	{837., 3214.}	89.6125	1.03536	newly 3 weeks cumul	25641.	3399.7	{1824., 5976.}	86.7412	0.918642	newly 5 weeks spec	20012.	4024.23	{1612., 7712.}
25	newly 3 weeks spec	12372.	4255.15	{4006., 4451.}	65.6066	0.463967	newly 3 weeks cumul	23919.	9433.08	{8958., 9806.}	60.5624	0.404392	newly 5 weeks spec	15457.	6650.63	{6359., 6941.}
26	newly 3 weeks spec	12283.	6392.44	{6241., 6547.}	47.957	0.283712	newly 3 weeks cumul	28429.	15390.9	{15168., 15598.}	45.8619	0.266527	newly 5 weeks spec	11730.	7747.36	{7592., 7889.}
27	newly 3 weeks spec	12409.	6299.68	{6132., 6469.}	49.233	0.294503	newly 3 weeks cumul	28757.	14836.4	{14574., 15108.}	48.4076	0.287455	newly 5 weeks spec	11908.	7980.1	{7822., 8128.}
28	newly 3 weeks spec	16959.	6847.28	{4369., 8768.}	59.6245	0.418231	newly 3 weeks cumul	28083.	12441.9	{8232., 15703.}	55.6959	0.373854	newly 5 weeks spec	18063.	11951.3	{8727., 14609.}
29	newly 3 weeks spec	12914.	6599.26	{5443., 7050.}	48.8984	0.293572	newly 3 weeks cumul	24569.	13451.7	{11268., 14362.}	45.2493	0.263328	newly 5 weeks spec	15388.	10126.9	{8852., 10536.}
30	newly 3 weeks spec	13125.	1805.07	{746., 2986.}	86.2471	0.934636	newly 3 weeks cumul	20142.	3476.74	{1637., 5538.}	82.7389	0.820427	newly 5 weeks spec	19690.	4225.74	{1375., 7190.}
31	newly 3 weeks spec	10370.	7425.45	{7285., 7558.}	28.3949	0.145092	newly 3 weeks cumul	26082.	19510.2	{19340., 19679.}	25.1966	0.126089	newly 5 weeks spec	9931.	7614.86	{7501., 7741.}
32	newly 3 weeks spec	12614.	9222.25	{9091., 9358.}	26.8888	0.136046	newly 3 weeks cumul	26998.	20683.7	{20408., 20928.}	23.3879	0.115723	newly 5 weeks spec	13001.	10606.	{10465., 10751.}
33	newly 3 weeks spec	20264.	9882.81	{9350., 10567.}	51.2297	0.312867	newly 3 weeks cumul	37971.	19308.5	{18062., 20741.}	49.1494	0.294721	newly 5 weeks spec	15166.	12923.8	{12687., 13222.}
34	newly 3 weeks spec	15602.	9706.97	{9482., 9861.}	37.7838	0.206157	newly 3 weeks cumul	34185.	22496.	{21546., 22827.}	34.1934	0.181816	newly 5 weeks spec	13645.	10448.9	{10300., 10609.}
35	newly 3 weeks spec	18683.	5606.73	{2752., 7544.}	69.9902	0.562539	newly 3 weeks cumul	29482.	9909.44	{5183., 13441.}	66.3882	0.50596	newly 5 weeks spec	19217.	11240.1	{6072., 14074.}
36	newly 3 weeks spec	11929.	4656.91	{4183., 5174.}	60.9614	0.412411	newly 3 weeks cumul	21930.	9724.04	{8720., 10722.}	55.6587	0.356604	newly 5 weeks spec	15024.	7872.7	{7356., 8520.}
37	newly 3 weeks spec	15965.	1199.21	{733., 1582.}	92.4885	1.14182	newly 3 weeks cumul	25084.	2586.55	{1707., 3318.}	89.6884	1.00047	newly 5 weeks spec	18951.	2330.51	{1274., 3143.}
38	newly 3 weeks spec	14686.	5546.36	{5417., 5648.}	62.2337	0.422967	newly 3 weeks cumul	31691.	12799.9	{12607., 12947.}	59.6102	0.393753	newly 5 weeks spec	13535.	7508.16	{7336., 7647.}
39	newly 3 weeks spec	14352.	7246.82	{6865., 7564.}	49.5065	0.297111	newly 3 weeks cumul	28679.	15722.2	{14919., 16462.}	45.1788	0.261406	newly 5 weeks spec	15042.	9781.13	{9560., 10006.}
40	newly 3 weeks spec	14498.	9161.88	{8950., 9322.}	36.8059	0.199371	newly 3 weeks cumul	31327.	20683.9	{19868., 21111.}	33.9744	0.180407	newly 5 weeks spec	13194.	10454.8	{10222., 10721.}
41	newly 3 weeks spec	13435.	1110.63	{330., 2192.}	91.7333	1.17188	newly 3 weeks cumul	19619.	2210.09	{819., 4084.}	88.735	1.01893	newly 5 weeks spec	23349.	2618.19	{571., 5820.}
42	newly 3 weeks spec	10315.	6554.82	{6342., 6711.}	36.4535	0.197033	newly 3 weeks cumul	25161.	16383.8	{15798., 16729.}	34.8841	0.186415	newly 5 weeks spec	10541.	7528.56	{7357., 7687.}
43	newly 3 weeks spec	12045.	6868.87	{6450., 7332.}	42.9733	0.244408	newly 3 weeks cumul	24152.	14739.7	{13826., 15859.}	38.9711	0.215051	newly 5 weeks spec	14237.	9654.58	{9326., 9968.}
44	newly 3 weeks spec	16196.	7426.66	{7187., 7677.}	54.1451	0.338787	newly 3 weeks cumul	32797.	15694.	{15148., 16061.}	52.148	0.320228	newly 5 weeks spec	14170.	10345.9	{10093., 10588.}
45	newly 3 weeks spec	16795.	6879.3	{6590., 7094.}	59.0396	0.387843	newly 3 weeks cumul	31747.	13974.	{13319., 14371.}	55.9833	0.356617	newly 5 weeks spec	14996.	10492.1	{10314., 10677.}
46	newly 3 weeks spec	15111.	6695.	{3454., 8714.}	55.6945	0.374808	newly 3 weeks cumul	25292.	11885.	{6583., 15240.}	53.0091	0.345084	newly 5 weeks spec	17991.	12388.5	{7203., 14513.}
47	newly 3 weeks spec	13356.	4278.57	{4156., 4394.}	67.9652	0.494532	newly 3 weeks cumul	27095.	9404.19	{9233., 9584.}	65.2918	0.459642	newly 5 weeks spec	14547.	6796.13	{6567., 6992.}
48	newly 3 weeks spec	14508.	5806.81	{5326., 6183.}	59.9751	0.399524	newly 3 weeks cumul	25267.	11448.3	{10236., 12256.}	54.6909	0.345624	newly 5 weeks spec	17877.	10011.3	{9749., 10355.}
49	newly 3 weeks spec	15110.	10240.3	{10068., 10443.}	32.2283	0.168994	newly 3 weeks cumul	34829.	24554.3	{24128., 25157.}	29.5004	0.151862	newly 5 weeks spec	12499.	10277.1	{10107., 10409.}
50	newly 3 weeks spec	13796.	8723.57	{8405., 8927.}	36.7674	0.199162	newly 3 weeks cumul	28249.	18991.6	{18123., 19437.}	32.7708	0.172582	newly 5 weeks spec	14245.	10771.5	{10600., 10927.}

1	52.1403	0.320114	newly 5 weeks cumul	57301.	22261.7	{21946., 22612.}	61.1496	0.410633	newly 55 weeks cumul	84027.	73463.4	{73040., 73873.}	12.5717	0.0583527
2	47.5235	0.284114	newly 5 weeks cumul	67910.	25688.9	{23494., 27677.}	62.1722	0.427086	newly 62 weeks cumul	97717.	92389.1	{92347., 92835.}	5.45241	0.0243796
3	33.5259	0.191521	newly 5 weeks cumul	61841.	34192.7	{18915., 42667.}	44.7088	0.275262	newly 64 weeks cumul	97783.	93243.9	{90706., 94153.}	4.64199	0.0207242
4	34.3187	0.182681	newly 5 weeks cumul	59339.	35099.4	{34104., 36349.}	40.8494	0.228209	newly 62 weeks cumul	94062.	91147.	{90988., 92249.}	3.09907	0.0138057
5	30.2748	0.178207	newly 5 weeks cumul	77798.	31975.1	{16197., 41571.}	58.8999	0.411621	newly 64 weeks cumul	97923.	91432.9	{82867., 93664.}	6.62777	0.0304796
6	33.5397	0.18616	newly 5 weeks cumul	74866.	36181.6	{25222., 45305.}	51.6715	0.331108	newly 63 weeks cumul	93450.	89605.3	{81532., 92593.}	4.11421	0.0188856
7	44.6405	0.256866	newly 5 weeks cumul	52923.	24485.8	{24149., 24758.}	53.7331	0.33475	newly 55 weeks cumul	88800.	77219.5	{76802., 77662.}	13.0411	0.0606901
8	29.5719	0.152319	newly 5 weeks cumul	65812.	32840.3	{31810., 33724.}	50.0999	0.302024	newly 62 weeks cumul	97109.	92382.2	{92141., 92608.}	4.86752	0.021672
9	77.7384	0.785864	newly 5 weeks cumul	78660.	9672.21	{2358., 16856.}	87.7038	1.01908	newly 64 weeks cumul	98833.	80833.8	{62924., 93404.}	18.2118	0.0923507
10	29.343	0.150983	newly 5 weeks cumul	52825.	33293.8	{31940., 34165.}	36.9734	0.200628	newly 60 weeks cumul	95503.	89025.2	{88071., 89620.}	6.78281	0.0305142
11	55.5129	0.352103	newly 5 weeks cumul	57151.	19975.	{19477., 20452.}	65.0488	0.456855	newly 59 weeks cumul	97210.	84105.8	{83818., 84375.}	13.4804	0.0628882
12	29.9068	0.154436	newly 5 weeks cumul	64453.	34746.8	{33174., 35541.}	46.0897	0.268473	newly 60 weeks cumul	95054.	89468.3	{89190., 89749.}	5.8764	0.0263032
13	36.809	0.199424	newly 5 weeks cumul	62511.	29206.3	{28653., 29853.}	53.2782	0.330542	newly 61 weeks cumul	96828.	91056.2	{90772., 91277.}	5.96091	0.0266925
14	63.147	0.433716	newly 5 weeks cumul	46832.	15961.	{15572., 16377.}	65.9187	0.467574	newly 54 weeks cumul	89533.	72604.7	{71699., 73740.}	18.9073	0.091042
15	73.2488	0.572842	newly 5 weeks cumul	59486.	12465.	{12126., 12784.}	79.0456	0.678812	newly 55 weeks cumul	91749.	73411.5	{72634., 74498.}	19.9866	0.0968568
16	44.0779	0.252486	newly 5 weeks cumul	50447.	26046.9	{25586., 26518.}	48.3679	0.287129	newly 58 weeks cumul	87287.	79177.5	{78535., 79642.}	9.29067	0.0423538
17	55.2387	0.385592	newly 5 weeks cumul	68260.	24111.9	{14064., 37736.}	64.6764	0.492563	newly 64 weeks cumul	92364.	88063.	{81837., 93633.}	5.87531	0.0272775
18	65.0992	0.500208	newly 5 weeks cumul	70235.	15447.4	{7910., 21258.}	78.0062	0.694732	newly 63 weeks cumul	98793.	87320.5	{72321., 93516.}	11.6127	0.0560233
19	60.9896	0.409178	newly 5 weeks cumul	54787.	18140.	{17088., 18740.}	66.8899	0.480409	newly 58 weeks cumul	96606.	84342.5	{84081., 84783.}	12.6943	0.0589788
20	37.4278	0.212998	newly 5 weeks cumul	72041.	31858.4	{24207., 44892.}	55.7773	0.370209	newly 63 weeks cumul	97397.	88458.2	{81292., 92607.}	9.17765	0.0426586
21	43.3251	0.280432	newly 5 weeks cumul	73114.	27430.	{15232., 37790.}	62.4833	0.46401	newly 64 weeks cumul	98246.	90033.9	{82307., 93705.}	8.35873	0.0388356
22	24.4646	0.121901	newly 5 weeks cumul	61831.	39978.2	{38813., 40846.}	35.3428	0.18947	newly 60 weeks cumul	92206.	90516.5	{89870., 91228.}	1.83228	0.00803684
23	20.2161	0.0981174	newly 5 weeks cumul	65072.	42067.2	{41303., 42795.}	35.3529	0.18949	newly 61 weeks cumul	83725.	89654.9	{88874., 90201.}	7.08253	0.0297121
24	79.8909	0.758646	newly 5 weeks cumul	69280.	10171.7	{4735., 18907.}	85.3179	0.8866	newly 63 weeks cumul	98228.	83889.2	{61056., 93449.}	14.5975	0.0733974
25	56.9733	0.366747	newly 5 weeks cumul	55191.	21602.9	{20680., 22532.}	60.8579	0.407729	newly 59 weeks cumul	91517.	79293.4	{78837., 80820.}	13.3566	0.0627519
26	33.9526	0.18019	newly 5 weeks cumul	53475.	30450.3	{30027., 30835.}	43.0569	0.244581	newly 57 weeks cumul	80102.	78142.4	{77856., 78408.}	2.44638	0.0107594
27	32.9854	0.173883	newly 5 weeks cumul	53686.	30204.8	{29710., 30681.}	43.7381	0.249817	newly 57 weeks cumul	90968.	80545.9	{80234., 80836.}	11.4569	0.0528471
28	33.8352	0.192406	newly 5 weeks cumul	68202.	34470.9	{23993., 42622.}	49.4577	0.313997	newly 64 weeks cumul	88415.	92661.3	{89854., 93981.}	5.86934	0.0251336
29	34.1894	0.182679	newly 5 weeks cumul	56007.	32364.9	{27523., 34088.}	42.2128	0.239578	newly 61 weeks cumul	96532.	91491.7	{89492., 92037.}	5.22141	0.0233112
30	78.5386	0.76144	newly 5 weeks cumul	60177.	10583.8	{4148., 17715.}	82.4122	0.832814	newly 64 weeks cumul	98710.	80545.1	{60074., 93592.}	18.4023	0.0939042
31	23.3223	0.11536	newly 5 weeks cumul	46868.	34875.4	{34558., 35132.}	25.5881	0.128365	newly 54 weeks cumul	76589.	77597.8	{77361., 77827.}	1.31717	0.00568171
32	18.4216	0.0884487	newly 5 weeks cumul	54036.	41836.6	{41480., 42213.}	22.5765	0.111137	newly 60 weeks cumul	93822.	89830.5	{89647., 89982.}	4.25438	0.0188815
33	14.7841	0.0696982	newly 5 weeks cumul	73450.	44741.6	{43217., 46786.}	39.0857	0.215822	newly 64 weeks cumul	96763.	90976.2	{90931., 91511.}	5.98034	0.0269774
34	23.4233	0.115929	newly 5 weeks cumul	63916.	43635.1	{42738., 44010.}	31.7306	0.1658	newly 60 weeks cumul	89965.	88811.6	{88580., 89321.}	1.28211	0.00560623
35	41.5098	0.263438	newly 5 weeks cumul	73185.	30012.3	{16335., 38741.}	58.9911	0.420436	newly 64 weeks cumul	94833.	90903.6	{82501., 93647.}	4.14348	0.0190184
36	47.5992	0.283795	newly 5 weeks cumul	52068.	23984.9	{21912., 26266.}	53.9354	0.340034	newly 60 weeks cumul	97587.	88409.8	{88472., 89693.}	9.40411	0.0432459
37	87.7024	0.93453	newly 5 weeks cumul	65780.	6610.76	{4064., 8680.}	89.9502	1.01676	newly 61 weeks cumul	98650.	78054.4	{68441., 82446.}	20.8774	0.103001
38	44.5278	0.255989	newly 5 weeks cumul	60924.	27017.4	{26672., 27369.}	55.6539	0.353169	newly 58 weeks cumul	87770.	79141.	{78839., 79393.}	9.83135	0.0449463
39	34.9745	0.187023	newly 5 weeks cumul	60279.	34436.3	{33207., 35552.}	42.8719	0.243357	newly 60 weeks cumul	93593.	89272.9	{88657., 89793.}	4.61588	0.0205293
40	20.7613	0.10113	newly 5 weeks cumul	59514.	41570.9	{40962., 41925.}	30.1494	0.155847	newly 61 weeks cumul	94658.	89067.3	{88364., 90613.}	5.9062	0.0264638
41	88.7867	1.0762	newly 5 weeks cumul	66595.	6583.93	{1804., 13684.}	90.1135	1.10465	newly 64 weeks cumul	95882.	77801.9	{56761., 93068.}	18.8566	0.0981989
42	28.5783	0.146243	newly 5 weeks cumul	46907.	31187.9	{30242., 31831.}	33.5112	0.17733	newly 55 weeks cumul	87719.	80025.1	{79652., 80363.}	8.77112	0.0398711
43	32.1867	0.168845	newly 5 weeks cumul	52694.	33041.1	{31599., 34750.}	37.2962	0.203007	newly 60 weeks cumul	95113.	89380.7	{88454., 90434.}	6.02681	0.0270105
44	26.9872	0.136683	newly 5 weeks cumul	64055.	35412.8	{34357., 36171.}	44.7149	0.257487	newly 60 weeks cumul	95957.	91202.7	{90867., 91445.}	4.95459	0.0220703
45	30.0338	0.155157	newly 5 weeks cumul	65154.	33611.4	{32662., 34279.}	48.4124	0.287557	newly 62 weeks cumul	96985.	92273.9	{92082., 92503.}	4.85754	0.0216264
46	31.1404	0.174889	newly 5 weeks cumul	63693.	34513.7	{18843., 42621.}	45.8125	0.282125	newly 64 weeks cumul	97957.	92820.	{90787., 93639.}	5.2441	0.0235182
47	53.2816	0.330624	newly 5 weeks cumul	57180.	21812.9	{21324., 22268.}	61.8522	0.418604	newly 59 weeks cumul	92154.	82864.3	{82250., 83296.}	10.0806	0.0461514
48	43.9988	0.252838	newly 5 weeks cumul	62499.	29668.7	{27832., 31098.}	52.5294	0.324912	newly 62 weeks cumul	95356.	92723.2	{92807., 93192.}	2.76103	0.0122469
49	17.777	0.0850331	newly 5 weeks cumul	62570.	45696.6	{45248., 46223.}	26.9673	0.136495	newly 60 weeks cumul	88106.	88210.6	{87729., 88558.}	0.298198	0.00129401
50	24.3842	0.121415	newly 5 weeks cumul	58206.	40129.3	{39285., 40645.}	31.0564	0.161544	newly 61 weeks cumul	94140.	90974.7	{90705., 91282.}	3.36236	0.0148552
